# Modified Significance Analysis of Microarrays in Heterogeneous Diseases

**DOI:** 10.3390/jpm11020062

**Published:** 2021-01-20

**Authors:** I-Shiang Tzeng

**Affiliations:** 1 Department of Research, Taipei Tzu Chi Hospital, Buddhist Tzu Chi Medical Foundation, New Taipei 231, Taiwan; istzeng@gmail.com; Tel.: +886-2-6628-9779 (ext. 3119); 2 Department of Statistics, National Taipei University, Taipei 237, Taiwan; 3 Department of Applied Mathematics, Department of Exercise and Health Promotion, Chinese Culture University, Taipei 111, Taiwan

**Keywords:** gene expression, heterogeneous data, significance analysis of microarrays

## Abstract

Significance analysis of microarrays (SAM) provides researchers with a non-parametric score for each gene based on repeated measurements. However, it may lose certain power in general statistical tests to correctly detect differentially expressed genes (DEGs) which violate homogeneity. Monte Carlo simulation shows that the “half SAM score” can maintain type I error rates of about 0.05 based on assumptions of normal and non-normal distributions. The author found 265 DEGs using the half SAM scoring, more than the 119 DEGs detected by SAM, with the false discovery rate controlled at 0.05. In conclusion, the author recommends the half SAM scoring method to detect DEGs in data that show heterogeneity.

## 1. Background

Microarray experiments are conducted for the detection of differentially expressed genes (DEGs), identification of genes with a specific function, and clarification of genetic interaction networks using series of data points. Microarray data are produced via replicates through two different scenarios [1]. One of these scenarios consists in comparative experiments that show gene expression levels in two different groups (“control” vs. “treatment”). Carefully constructed comparative experiments can precisely detect DEGs between the two different groups. Another scenario could be that uncertainties in microarray data may arise from various sources, including measurement and data preprocessing [2] (i.e., non-biological variation during experimentation). For example, fold change is a rough method that is often used to distinguish changes in the expression levels of individual genes in a microarray [3]. Next-generation sequencing (NGS) has become a widely used tool, fueling a revolution in biomedical sciences by addressing the need to generate inexpensive, reproducible, and high-throughput nucleic acid sequence data [4,5]. The pattern of gene expression in a cell/tissue can broadly reflect its functional state. NGS-based expression profiling by sequencing of RNA (i.e., RNA-Seq) encompasses quantitative gene expression profiling and the discovery of novel transcribed sequences [6]. In addition, differential expression (DE) analysis is commonly used to compare the transcriptomes of two or more groups of samples. Fortunately, statistical analysis can be used to distinguish true changes from random variation by the calculation of significance values (i.e., *p*-values).

Various statistical approaches can be used to determine whether the null hypothesis is acceptable if the population or distribution is the only sampling source for two samples. In the last 15 years, innovative alternatives that rely upon either parametric or nonparametric approaches have been developed by many researchers. For example, the Wilcoxon rank-sum test or rank products for non-parametric tests have been applied extensively to microarray data [7,8,9]. Student’s *t*-test is one of the parametric tests used to analyze microarray data after verifying certain assumptions (“normality” and “homogeneity”). Repeated experiments were limited due to the great expense of experiments in the early period, so alternative approaches relying upon Bayesianism were developed, using small samples to calculate *p*-values by an empirical Bayes method [10,11]. The count data generated from digital gene expression experimentation, such as serial analysis of gene expression and RNA-seq, demonstrate more variance than is expected from a Poisson distribution model (overdispersion) [12], leading to an increase in type I error (false positives) in DE analysis. This observed overdispersion should be compensated prior to DE analysis because traditional DE algorithms, such as Student’s *t*-test and analysis of variance, assume normally distributed data. Several software packages can perform this task by utilizing different methodologies; these include DESeq [13], edgeR [14], Cuffdiff2 [15], and linear models for microarray analysis (LIMMA) [11]. After evaluating various methods, I also settled on LIMMA for DE analysis. The flexibility in the LIMMA package [16] allows us to model many different experimental factor configurations, minimize type I errors, and permit the correction of experimental batch factors. The final steps in DE analysis are to filter the data for fold change and determine the statistical significance. I initially set thresholds to twofold or greater change and a false discovery rate (FDR) of less than 5%. Furthermore, state-of-the-art algorithms like edgeR, DESeq2 [17], Sleuth [18], and so on. In particular, Sleuth is a method which is capable of including the technical errors of newer aligning algorithms into the model. However, violation of the homogeneity assumption may reduce the power of general statistical tests in detecting DEGs in heterogeneous diseases [19]. The definition of a heterogeneous disease is various morphological features and clinical behaviors exhibited due to a multitude of etiological entities. Heterogeneous diseases include acute lymphoblastic leukemia [20], primary thyroid lymphoma [21], otosclerosis [22], and colorectal cancer [23]. Heterogeneous diseases may cause a gene to be overexpressed in some cases, but expressed normally or underexpressed in the remaining cases. The case group exhibits higher variance than the control group due to multimodality. The traditional Student’s *t*-test could not be used to detect the gene in such a scenario. However, the “half Student’s *t*-test” may have greater power under conditions of heterogeneity.

Significance analysis of microarrays (SAM) prepares scores for each gene depending on changes in gene expression connected to the standard deviation derived from repeated measurements [3]. To identify DEGs from heterogeneous data, the “half SAM” is proposed. The half SAM is adapted to adjust the moderated t statistic for heterogeneous data from the population distribution. The proposed approach is derived from components of the SAM and half Student’s *t*-test [24]. The null compliance hypothesis asserts that gene expression data from the case and control groups have equal distribution (i.e., the means, variances, or both are equal). To my knowledge, the null compliance hypothesis means no meaningful clinical difference between the two groups in terms of distribution compliance with the source population. The alternative hypothesis asserts that the means, variances, or both differ between the two groups. In this study, it was assumed that the mean response in the case group shows an increase in variability, accordingly.

I performed a Monte Carlo simulation to determine the statistical features for various methods, including Student’s *t*-test [25], half Student’s *t*-test [24], SAM [3], and half SAM, and a gene expression dataset of colon cancer was analyzed to provide a demonstration [26]. The implicit assumptions, structure of the proposed method, complexity of the computation, and usability for microarray data [27] are discussed in this study.

## 2. Materials and Methods

For a gene expression dataset, the terms $n_{1}$, ${\bar{X}}_{1}$, and $s_{1}$ for the case group denote the sample size, sample mean, and sample standard deviation, respectively. The terms $n_{0}$, ${\bar{X}}_{0}$, and $s_{0}$ denote the same for the control group. The conventional Student’s *t*-test, denoted $t_{s}$ and used to detect DEGs, is as follows:

$$t_{s}=\frac{\bar{X_{1}}-\bar{X_{0}}}{s_{p}\sqrt{\frac{1}{n_{1}}+\frac{1}{n_{0}}}}$$

where $s_{p}=\sqrt{\frac{\left(n_{1}-1\right)s_{1}{}^{2}+\left(n_{0}-1\right)s_{0}{}^{2}}{n_{1}+n_{0}-2}}$ defines the pooled standard deviation. The $t_{s}$ statistic obeys a Student’s *t* distribution encompassing $n_{1}+n_{0}-$2 degrees of freedom (df) under assumption of normality.

SAM is a popular method for modification of the conventional *t*-statistic. The modified statistic is defined as

$$d_{SAM}=\frac{\bar{X_{1}}-\bar{X_{0}}}{s_{p}\sqrt{\frac{1}{n_{1}}+\frac{1}{n_{0}}}+c_{0}}$$

where $c_{0}$ is used to guarantee the difference in the coefficient of variation of $d_{SAM}$ to be minimized within classes of genes under approximately equivalent variance. 

Recently, the half Student’s *t*-test, using the standard deviation of the control group, was used to solve the heterogeneity issue. The half Student’s *t*-test statistic is defined as $t_{h}$ as follows:

$$t_{h}=\frac{\bar{X_{1}}-\bar{X_{0}}}{s_{0}\sqrt{\frac{1}{n_{1}}+\frac{1}{n_{0}}}}$$

The normality assumption for $t_{h}$ obeys a Student’s *t* distribution encompassing $n_{0}-1$ df when the null hypothesis is correct.

**Figure 1:**
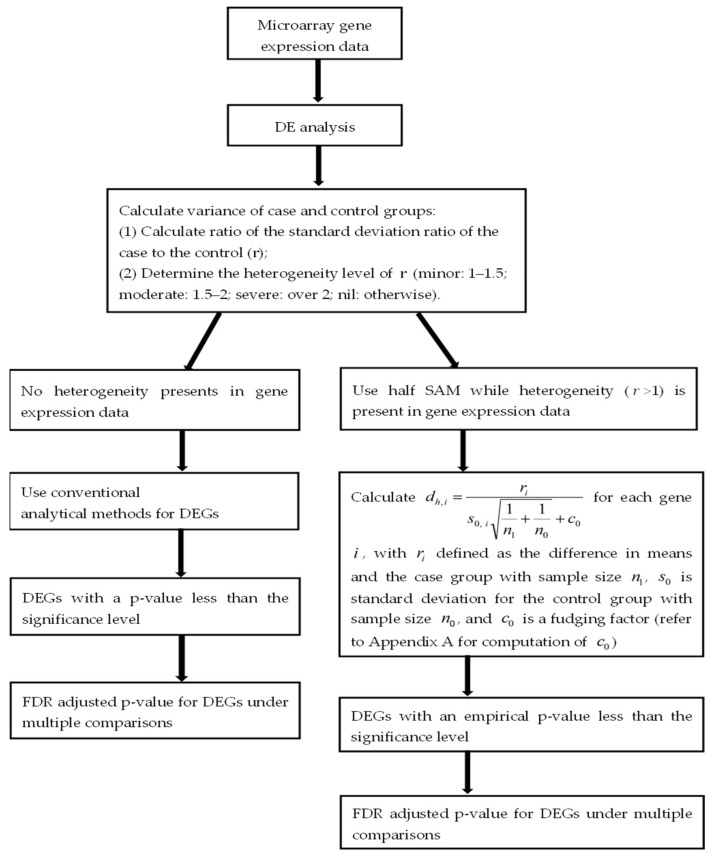
Workflow to identify differentially expressed genes in this study.

I modified the scoring for the SAM and the half SAM as follows:

$$d_{h}=\frac{\bar{X_{1}}-\bar{X_{0}}}{s_{0}\sqrt{\frac{1}{n_{1}}+\frac{1}{n_{0}}}+c_{0}}$$

where $d_{h}$ involves $s_{0}$ only. For the stable adjusted term $c_{0}$, the score $d_{h}$ does not follow a Student’s *t* distribution. (For more details on the calculation of $c_{0}$, refer to the Appendix A) Figure 1 illustrates the workflow of the half SAM calculation in this study.

### 2.1. Monte Carlo Simulation

The free statistical software R [28] was used for testing and analysis in this study. One thousand genes with small sample sizes of 20 ($n_{0}=n_{1}=10$) were simulated. The term md denotes the difference in the means between the two groups and was set to 0, 10, and 15. Notation r denotes the standard deviation ratio of the case to the control and was set to 1, 1.5, and 2. Moreover, $s_{0}$ was set to 15. Gene expression levels were assumed to follow a normal distribution. The normality assumption is usually applicable for empirical gene expression data [24]. Three scenarios following a non-normal distribution were considered: (1) a symmetric and non-normal distribution; (2) a right-skewed distribution; and (3) a left-skewed distribution. The uniform distribution was used as the symmetric and non-normal distribution. The Gamma distribution was used as the right-skewed distribution. The Gamma distribution multiplied by −1 and added with double the expected value of the initial Gamma distribution was used as the left-skewed distribution. 

In general, “heterogeneous data” usually refers to the situation where the data consist of multiple subgroups of patients with different characteristics. I also considered a simulation scenario where the gene expression for the case group is generated from a mixed distribution, with different mixture components representing the heterogeneity of individuals’ expressions in the case group.

For each setting, Student’s *t*-test, SAM scoring, and half SAM scoring were performed under 1,000,000 simulations. Details on the simulation procedure are provided in previous publications [8,19].

### 2.2. An Example for Demonstration

Alon et al. provided a colon cancer dataset [26] which I analyzed to provide a demonstration in this study. The colon cancer dataset (downloadable at http://genomics-pubs.princeton.edu/oncology/) is a set including measurements of the expression of 2000 genes from 62 samples. In addition, the data comprise 40 colon cancer tissue case samples and 22 healthy tissue control samples. Details on the arrays of oligonucleotides providing colon cancer data are provided in my previous publication [8].

## 3. Results

### 3.1. Simulation Results

Type I error and power as a percentage calculated at a significance level of 0.05 are presented in Table 1. Student’s *t*-test, half Student’s *t*-test, half SAM, and SAM maintained type I error rates of about 0.05 for all settings of distributions and at each significance level with small sample sizes ($n_{0}=n_{1}=10$) for the two groups. However, the type I error rates of the $t_{s}$, $d_{SAM}$, and $d_{h}$ statistics were much lower than the significance levels for small sample sizes under a left-skewed distribution. 

**Figure 2:**
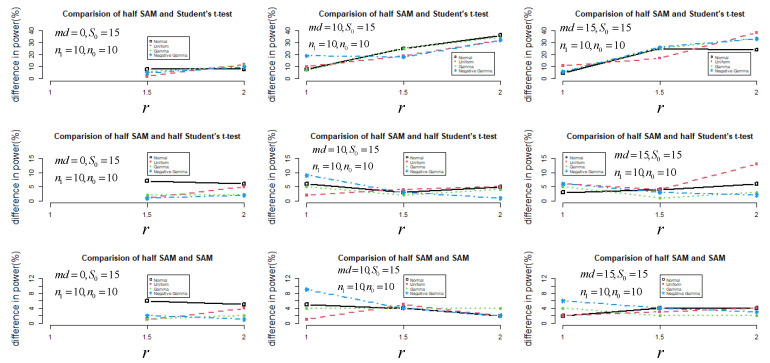
The difference in power (%) between half SAM and SAM (or half Student’s *t*-test and Student’s *t*-test) under small sample sizes for $n_{0}=n_{1}=10$ with $s_{0}$ set to 15.

In Figure 2, it can be seen that the power performance of half SAM and SAM was similar under non-normal distribution scenarios, especially for r≤1.5. Besides this, half SAM was more powerful than SAM overall when r≥1.5 and md>0. The maximal difference in power between half SAM and SAM was about 9% under skew-to-left distribution scenarios with md=15 and r=1. Note that both score tests ($d_{h}$ and $d_{SAM}$) had some power for identifying differences between variances when md=0, with power increasing as r increased. However, when r increased for md=15, both score tests’ power marginally decreased. 

**Figure 3:**
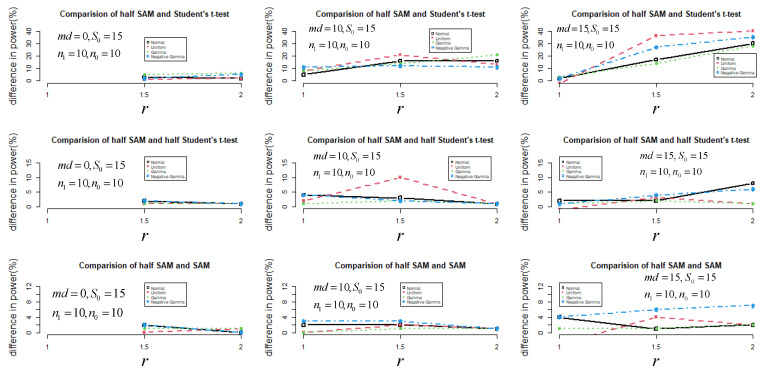
The difference in power (%) between half SAM and SAM (or half Student’s *t*-test and Student’s *t*-test) after controlling the FDR at 0.05.

Since 1000 genes in total with small sample sizes ($n_{0}=n_{1}=10$) were simulated, I constructed a comparison of control of the FDR based on different statistics ($t_{s}$, $d_{s}$, and $d_{h}$) to declare the statistical power performance in a Monte Carlo simulation (Figure 3). After controlling the FDR at 0.05, half SAM was still more powerful than the other statistics under a non-normal distribution when md=1 and r=1. 

Table 2 presents the respective numbers (percentages) of DEGs identified by Student’s *t*, SAM score, half Student’s *t*, and half SAM score. Four significance levels—0.05, 0.01, 0.005, and 0.001—were examined. It should be remembered that the scorings of $d_{SAM}$ and $d_{h}$ did not follow a Student’s *t* distribution. For a fair comparison, I adopted bootstrapping to evaluate the empirical performance of permutation testing through statistics [29] ($t_{s}$, $d_{SAM}$, $t_{h}$, and $d_{h}$). I found that the SAM scoring method detected a similar number of DEGs to Student’s *t*-test, for all significance levels. However, the half SAM score detected more DEGs than Student’s *t* at each significance level. 

Moreover, I also considered a simulation of the gene expressions generated from a mixed distribution (details not shown). I found that half SAM was still more powerful than the other statistics (Supplementary Materials Table S1).

### 3.2. Main Results for Colon Cancer Data

A total of 2000 genes from these datasets were considered for multiple comparison testing. The FDR [30] was controlled at 0.05. It was found that the half SAM score could detect 265 DEGs when the FDR was controlled at 0.05—more than the 119 DEGs detected by the SAM score.

### 3.3. Main Results for RNA-Seq Data

**Figure 4:**
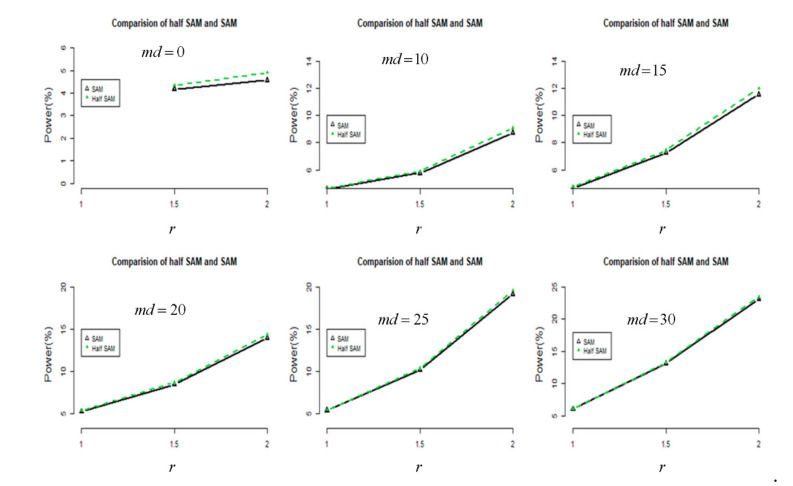
A comparison in terms of power (%) between half SAM and SAM using RNA-Seq data for analysis. (Black: SAM; Green: Half SAM)

Because RNA-Seq experiments are a more common approach for transcriptome profiling, I performed a comparison of the proposed half SAM and SAM scores in DE using the RNA-Seq data from Himes et al. [31]. A total of 33,469 treated genes from airway datasets, extracted if assay of the genes was greater than 0 for summation of the count of eight sequence segments (SRR1039508, SRR1039509, SRR1039512, SRR1039513, SRR1039516, SRR1039517, SRR1039520, and SRR1039521) for comparison, with the FDR controlled at 0.05. The analysis results (refer to Figure 4) show that the half SAM score is more powerful than the SAM score when utilized with RNA-Seq airway data [31]. We may attribute this to the heterogeneity of airway data. These results indicate a greater impact due to the improved performance of the “half SAM” method when analyzing any publicly available RNA-Seq dataset. 

## 4. Discussion

There are two main methods for generating a whole transcriptome gene expression profile of tissues or cultures, namely, expression microarrays and next-generation RNA-Seq. The prevalence of microarrays has been steadily declining since its heyday, after it was developed in 1995. RNA-Seq has been widely used in the past decade, and it continues to be popular. Considering the role and advantages of RNA-Seq, such as detection of novel, unannotated genes, this is not surprising. A mixed model [32] approach which follows empirical Bayes approaches [33] and SAM can be used in practice for real data. Specifically, an empirical Bayes approach is adopted in LIMMA [11] to estimate a hyperparameter of the denominator. Theoretically, LIMMA [11] uses an empirical Bayes moderated *t*-test, computed for each probe, which is similar to a *t*-test, except that the standard errors are shrunk towards a common value. To my information, SAM handled RNA-Seq data [34] was competitive to popular parametric methods (i.e., edgeR and DESeq). It is worth mentioning that machine learning methods have also been widely applied to microarray data and RNA-Seq data [35]. For example, InfoGain [36] feature selection may be more powerful and robust in the detectability of DEGs.

**Figure 5:**
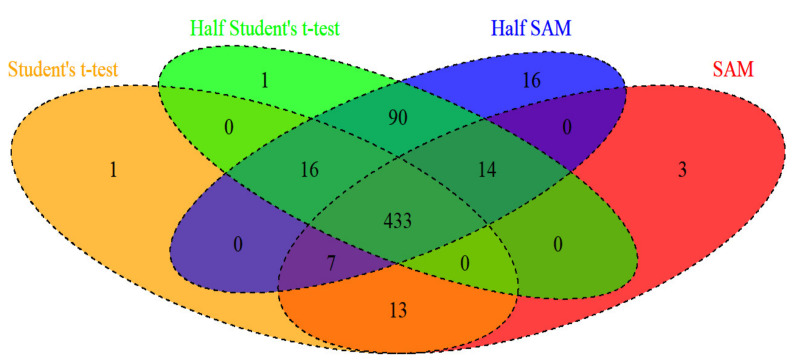
Venn diagram of differentially expressed genes detected by the various test methods in colon cancer data at a significance level of 0.05.

This study found that the half SAM score test fairly maintains the nominal α level for use on data with a normal or skewed distribution when the standard deviation ratio is large enough (i.e., r>1), and that the half SAM score is more powerful than the SAM score. This indicates that the half SAM score test is applicable for studying arrays of oligonucleotide data of heterogeneous diseases. In fact, more than one entity is present in a heterogeneous disease, causing various clinical presentations or etiologies which may lead to the standard deviation ratio being larger than 1 (i.e., when the standard deviation of case samples is greater than that of the control samples). Moreover, the percentage of DEGs seems to rise considerably with both the half Student’s *t*-test and half SAM score (Table 2). Both the half Student’s *t*-test [24] and half SAM score were proposed as modifications of traditional approaches (i.e., Student’s *t*-test and SAM score) for heterogeneous diseases. In addition, I provided Venn diagram (Figure 5) of DEGs under four test methods (at significance level of 0.05) in colon cancer data. Proposed half SAM detected at least 97% (i.e., 453/470 = 0.9702) overlap in DEGs match the baseline (set as the Student’s *t*-test marked yellow in Figure 5). The half SAM detected the highest number of novel DEGS (i.e., 16) compared to other methods. For sensitive examination, I constructed similar Monte Carlo simulations, but with unequal sample sizes (i.e., $n_{0}\neq n_{1}$). I found that the SAM score and other tests can still maintain quite low type I error rates under all situations with unequal sample sizes. The half SAM score also has more power than other tests under situations with unequal sample sizes.

SAM can be used to detect genes that show significantly different expression between sets of samples (“control” and “treatment”). In this study, SAM was implemented for two-class unpaired analysis (“control” vs. “treatment”). For each gene, I computed score values ($d_{SAM}$ or $d_{h}$, analogous to Student’s *t*). Using a permutation test procedure, I calculated the number (percentage) of DEGs identified by each of the investigated test methods. The rationale behind the use of SAM is that any genes designated as significant based on the randomized data are being identified purely by chance. 

Note that the microarray data in the original scale are right-skewed. Based on simulation results, researchers may analyze microarray data in the original scale (corresponding to a non-normal distribution or mixed scenario) or log-transformed scale data (corresponding to a normal distribution scenario) under the proposed DEG method with greater statistical power. Therefore, I clarified the half SAM method applied on the original-scale data or log-transformed data. 

SAM uses the principle of permutation to use a given sample to derive the theoretical sampling distribution of the test statistic. In practical problems, the test statistic’s exact sampling distribution is often not available, and the approximate sampling distribution can be estimated by a random number (or combination) of a large number of repetitions based on the sample. In practice, it is difficult for researchers to determine an appropriate test statistic to detect DEGs. For example, I found 389 DEGs under the LIMMA package [16] installed by R software with colon cancer data. The number of DEGs varied with different design matrix settings. If researchers are not familiar with design matrix settings [37] of LIMMA [11] for gene expression analysis, they may receive overestimated results (i.e., almost all genes, about 1,898 DEGs, showing as significantly differentially expressed). Returning to practice in this study, the author suggests that researchers use both the SAM and the half SAM to compare results when gene expression data show heterogeneity. Moreover, when the heterogeneity of the gene expression data is undetermined, the author suggests that researchers should not use both score tests simultaneously in the beginning. 

There are some limitations in this study. First, anyone can claim superiority only by improving sensitivity, but advice on limited experiments was not modified in the statistical approach. Conclusion may be logically inferred from Figure 4 of Alon’s [26] research. The devices used in Alon’s study [26] for data acquisition were not used during the heyday of microarrays. Regarding the device (the Affymetrix GeneChip), various preprocessing methods such as robust multi-array averaging [38] and the multiplicative model-based expression index [39] have been proposed to obtain a gene expression matrix from probe-level data (i.e., CEL files which created by Affymetrix DNA microarray image analysis software). The result varies considerably depending on which preprocessing method is used [40]. Second, the author focused on a comparison of t statistic-like approaches (i.e., Student’s *t*, SAM score, half Student’s *t*, and half SAM score) for fairness. Finally, the author acknowledges that functional annotation of these 146 DEGs (i.e., 265 − 119 = 146 under the FDR in Table 2) would show whether half SAM is able to detect genes implicated in different biological pathways or if they are associated with the same pathways identified by the other methods (i.e., SAM or half Student’s *t*).

## 5. Conclusions

Microarray experiments are conducted for the detection of different gene expression levels to target pathogenic genes for diseases. However, they may lose certain power when used with general statistical tests to adjustably detect DEGs which violate homogeneity. The half SAM score could identify 265 DEGs, more than the 119 DEGs detected by SAM, when the FDR was controlled at 0.05. The half SAM scoring method could be applicable for the identification of DEGs in heterogeneous diseases. In conclusion, the author recommends the half SAM scoring method to detect DEGs in data that show heterogeneity.

## Figures and Tables

**Table 1:** Type I error rates and statistical power for Student’s *t*-test, significance analysis of microarrays (SAM), and half SAM in normal and non-normal distributions.

Power % (Type I Error)	$n_{0}\;=\;n_{1}\;=\;10$ & md = 0				$n_{0}\;=\;n_{1}\;=\;10$ & md = 10				$n_{0}\;=\;n_{1}\;=\;10$ & md = 15			
	*t*-Test	Half *t*-Test	SAM	Half SAM	*t*-Test	Half *t*-Test	SAM	Half SAM	*t*-Test	Half *t*-Test	SAM	Half SAM
Normal distribution												
r= 1	(0.02)	(0.05)	(0.04)	(0.05)	28	30	31	36	60	62	63	65
r= 1.5	4	5	6	12	14	36	35	39	39	60	60	64
r= 2	7	9	10	15	8	39	42	44	28	46	48	52
Non-normal distribution												
r= 1	(0.05)	(0.05)	(0.04)	(0.04)	28	36	37	38	55	60	64	66
r= 1.5	8	9	9	10	19	34	33	38	38	51	52	55
r= 2	7	14	15	19	14	41	44	46	19	44	53	57
Skew-to-right distribution												
r= 1	(0.03)	(0.05)	(0.03)	(0.04)	28	30	31	35	46	47	48	52
r= 1.5	7	11	12	13	17	40	38	42	37	61	60	62
r= 2	4	13	13	15	9	39	39	43	24	54	55	57
Skew-to-left distribution												
r= 1	(0.03)	(0.04)	(0.03)	(0.03)	21	31	31	40	55	55	55	61
r= 1.5	6	10	9	11	20	35	34	38	39	62	61	65
r= 2	5	13	14	15	12	43	42	44	30	61	60	63

**Table 2:** Number (percentage) of differentially expressed genes detected by the various test methods in colon cancer data.

	Test Methods			
	Student’s *t*-Test	SAM Score	Half Student’s *t*-Test	Half SAM Score
Level of significance				
0.05	470(23.50%)	470(23.50%)	554(27.70%)	576(28.80%)
0.01	239(11.95%)	240(12.00%)	300(15.00%)	334(16.70%)
0.005	179(8.95%)	177(8.85%)	250(12.50%)	280(14.00%)
0.001	74(3.70%)	85(4.25%)	141(7.05%)	178(8.90%)
FDR				
0.05	107(5.35%)	119(5.95%)	216(10.3%)	265(13.25%)

## Data Availability

The data that support the findings of this study are available at http://genomics-pubs.princeton.edu/oncology/, https://www.ncbi.nlm.nih.gov/geo/query/acc.cgi?acc=GSE52778.

## Supplementary Materials

The following are available online at https://www.mdpi.com/2075-4426/11/2/62/s1. Table S1: Type I error rates and statistical power for Student’s *t*-test, SAM, and half SAM in mixed normal and mixed non-normal distributions.

## Appendix A

Computation of $c_{0}$.

Tusher et al., (2001) proposed SAM for finding significant genes in a set of microarray experiments. SAM produces a statistic, $d_{SAM,i}=\frac{r_{i}}{S_{i}+c_{0}}$, for each gene i, which measures the strength of the association between gene expression and the response variable. For two groups and unpaired data, $r_{i}$ is defined as the difference in means, and $S_{i}$ is calculated by the pooled standard deviation, $S_{P}$. For each gene i, $S_{i}$ equals to $s_{p}\sqrt{\frac{1}{n_{1}}+\frac{1}{n_{0}}}$, $s_{p}=\sqrt{\frac{\left(n_{1}-1\right)s_{1}{}^{2}+\left(n_{0}-1\right)s_{0}{}^{2}}{n_{1}+n_{0}-2}}$, $S_{1}$, is the standard deviation for the case group with sample size $n_{1}$, $S_{0}$ is the standard deviation for the control group with sample size $n_{0}$, and $c_{0}$ is a fudging factor. The modified SAM statistic is presented as $d_{h,i}=\frac{r_{i}}{s_{0,i}\sqrt{\frac{1}{n_{1}}+\frac{1}{n_{0}}}+c_{0}}\;$ of gene i using the sample standard deviation of the control group only.

1. Let $d_{h,i}^{a}=\frac{r_{i}}{s_{0}\sqrt{\frac{1}{n_{1}}+\frac{1}{n_{0}}}+c^{a}}$ and let $c^{a}$ be the α^th^ percentile of the $s_{0}\sqrt{\frac{1}{n_{1}}+\frac{1}{n_{0}}}$ values.
2. Compute $c^{a}$ for α∈ (0, 0.01, 0.02, …, 1.0) for the 100 quantiles, in turn, of the $s_{0}\sqrt{\frac{1}{n_{1}}+\frac{1}{n_{0}}}$ values denoted by $q_{1}$ < $q_{2}$ < … < $q_{100}$.
3. Consider $\alpha^{\prime }\in$ (0, 0.05, 0.10, …, 1.0) for the 20 quantiles in turn.
  (1) Let $v_{j}$ = mad($d_{h,i}^{d}$|$s_{0,i}\sqrt{\frac{1}{n_{1}}+\frac{1}{n_{0}}}\in$ [$q_{j}$, $q_{j+1}$)) divided by 0.64, j=1,2,…,n where mad is defined as the median absolute deviation from the median.
  (2) Let the coefficient of variation of the $v_{j}$ values be denoted as $\mathrm{cv}\left(\alpha^{\prime }\right)$.
4. Determine ${\hat{c}}_{0}$ according to certain $c^{\hat{\alpha }}$ via the criterion $\hat{\alpha }$ = argmin [$\mathrm{cv}\left(\alpha^{\prime }\right)$]. The fudging factor $c_{0}$ is determined by the value ${\hat{c}}_{0}$ in the end.
