# Supplementary material for: Modified Significance Analysis of Microarrays in Heterogeneous Diseases
Source: J Pers Med. 2021 Jan 20;11(2):62. doi: 10.3390/jpm11020062 (PMC7909396; doi:10.3390/jpm11020062)
Supplement: Supplementary file 1 [file jpm-11-00062-s001.pdf]

Table S1.Type I error rates and statistical power for Student's *t*-test, SAM, and half SAM in mixed normal and mixed non-normal distributions

Power % (Type I error)	$n_0 = n_1 = 10$ & $md = 0$				$n_0 = n_1 = 10$ & $md = 10$				$n_0 = n_1 = 10$ & $md = 15$			
	***t*-test**	**SAM**	**Half SAM**	**Difference**	***t*-test**	**SAM**	**Half SAM**	**Difference**	***t*-test**	**SAM**	**Half SAM**	**Difference**
**Mixture normal distribution**												
$r = 1$	(0.03)	(0.06)	(0.06)	—	22	31	33	2[11]	36	45	47	2[11]
$r = 1.5$	6	8	9	1[3]	16	22	25	3[9]	28	40	44	4[16]
$r = 2$	8	10	13	3[5]	11	23	27	4[16]	21	39	43	4[22]
**Mixture non-normal distribution**												
$r = 1$	(0.04)	(0.04)	(0.05)	—	14	19	21	2[7]	30	38	41	3[11]
$r = 1.5$	6	9	11	2[5]	11	22	25	3[14]	24	42	44	2[20]
$r = 2$	7	16	18	4[9]	7	26	28	2[21]	14	41	44	3[30]
**Mixture skew-to-right distribution**												
$r = 1$	(0.03)	(0.02)	(0.03)	—	24	32	33	1[9]	36	41	46	5[10]
$r = 1.5$	4	9	10	1[6]	12	22	24	2[12]	20	39	41	2[21]
$r = 2$	3	9	12	3[9]	5	21	23	2[18]	18	42	43	1[25]
**Mixture skew-to-left distribution**												
$r = 1$	(0.04)	(0.03)	(0.04)	—	18	24	25	1[7]	25	35	39	4[14]
$r = 1.5$	4	11	12	1[8]	17	34	37	3[20]	22	42	44	2[22]
$r = 2$	2	10	11	1[9]	11	23	25	2[14]	19	43	44	1[25]

Note: Power difference=Half SAM-SAM. Use notation "( )" for presentation of type I error. Use notation "[ ]" for presentation at power difference between half SAM and Student's *t*-test.

#0.3*N(mu1,sd1)+0.5*N(mu2,sd2)+0.2*N(mu3,sd3), where mu1=110, sd1=15; mu2=100, sd2=20, mu3=115, sd3=25.

#0.3*Uniform(a1,b1)+0.5*Uniform(a2,b2)+0.2*Uniform(a3,b3), where settings of a1, b1 satisfy mean and sd of Uniform(a1,b1) as 110 and 15, respectively; settings of a2, b2 satisfy mean and sd of Uniform(a2,b2) as 100 and 20, respectively; settings of a3, b3 satisfy mean and sd of Uniform(a3,b3) as 115 and 25, respectively

#0.3*Gamma(alpha1,beta1)+0.5*Gamma(alpha2,beta2)+0.2*Gamma(alpha3,beta3), where settings of alpha1, beta1 satisfy mean and sd of Gamma(alpha1,beta1) as 110 and 15, respectively; settings of alpha2, beta2 satisfy mean and sd of Gamma(alpha2,beta2) as 100 and 20, respectively; settings of alpha3, beta3 satisfy mean and sd of Gamma(alpha3,beta3) as 115 and 25, respectively
